# Kinetics of Procalcitonin, CRP, IL-6, and Presepsin in Heart Transplant Patients Undergoing Induction with Thymoglobulin (rATG)

**DOI:** 10.3390/jcm14155369

**Published:** 2025-07-29

**Authors:** Lorenzo Giovannico, Vincenzo Ezio Santobuono, Giuseppe Fischetti, Federica Mazzone, Domenico Parigino, Luca Savino, Maria Alfeo, Aldo Domenico Milano, Andrea Igoren Guaricci, Marco Matteo Ciccone, Massimo Padalino, Tomaso Bottio

**Affiliations:** 1 Cardiac Surgery Unit, Department of Precision and Regenerative Medicine and Ionian Area (DiMePRe-J), University of Bari Medical School, Piazza Giulio Cesare 11, 70124 Bari, Italy; lorenzo.giovannico@policlinico.ba.it (L.G.); giuseppefischetti23@gmail.com (G.F.); federica.mazzone95@gmail.com (F.M.); domenicoparigino@gmail.com (D.P.); lucasavino2905@gmail.com (L.S.); malfeo253@gmail.com (M.A.); aldo.milano@uniba.it (A.D.M.); massimo.padalino@uniba.it (M.P.); 2 University Cardiology Unit, Interdisciplinary Department of Medicine, University of Bari Aldo Moro, 70121 Bari, Italy; vincenzoezio.santobuono@uniba.it (V.E.S.); andrea.guaricci@uniba.it (A.I.G.); marcomatteo.ciccone@uniba.it (M.M.C.)

**Keywords:** Procalcitonin (PCT), Presepsin, Interleukin-6 (IL-6), C-reactive protein (CRP), Rabbit Anti-Thymocyte Globulin (rATG), heart transplantation, inflammatory biomarkers

## Abstract

**Background/Objectives**: Heart transplantation (HTx) is a lifesaving procedure for end-stage heart failure patients; however, postoperative infections remain a major challenge due to immunosuppressive therapy and surgical complications. Traditional biomarkers such as C-reactive protein (CRP) and procalcitonin (PCT) have limitations in distinguishing infections from systemic inflammatory response syndrome (SIRS). Emerging markers such as Presepsin and interleukin-6 (IL-6) may improve diagnostic accuracy. This study aimed to evaluate the kinetics and reliability of these four inflammatory biomarkers in heart transplant recipients in the immediate postoperative period. **Methods**: This retrospective observational study included 126 patients who underwent HTx at Policlinic of Bari between January 2022 and November 2024. Patients were categorized into infected (*n* = 26) and non-infected (*n* = 100) groups based on clinical and microbiological criteria. Biomarkers (CRP, PCT, Presepsin, and IL-6) were measured preoperatively and on postoperative days (PODs) 1, 2, 3, 4, 5, and 10. Statistical analyses included the Mann–Whitney U test and logistic regression to identify the independent predictors of infection. **Results**: CRP and PCT levels differed significantly between the groups only on day 10, limiting their use as early infection markers. In contrast, Presepsin levels were significantly elevated in infected patients from day 1 (*p* < 0.001), whereas IL-6 levels showed significant differences from day 3 onward. Presepsin showed the strongest association with infection in the early postoperative phase. **Conclusions**: Presepsin and IL-6 outperformed CRP and PCT in detecting early postoperative infections in heart transplant recipients. Their early elevation supports their use as reliable markers for guiding timely clinical intervention and improving patient outcomes. Further research is needed to validate these findings in larger cohorts and with different immunosuppressive regimens.

## 1. Introduction

Heart transplantation (HTx) is one of the most complex and demanding procedures in cardiac surgery. It is considered a lifesaving treatment for patients with end-stage heart failure who are refractory to other therapeutic interventions [1,2,3,4,5,6]. Although surgical techniques for HTx have advanced significantly over the past decades, the management of postoperative complications remains a challenge. Among the most significant complications are infections, which continue to be the leading cause of morbidity and mortality in heart transplant recipients [7,8,9,10,11,12]. Susceptibility to infection in HTx patients is multifaceted. It arises from a combination of factors, including the invasive nature of the surgery, use of immunosuppressive therapies to prevent graft rejection, and compromised physiological state due to the underlying cardiac condition. Immunosuppressive drugs, such as thymoglobulin (anti-thymocyte globulins, ATG), are essential for preventing acute and chronic rejection of the transplanted organ, but they also suppress the ability of the immune system to respond to pathogens [13,14,15,16,17,18]. ATG is an infusion of antibodies derived from horse or rabbit serum that target human T cells and their precursors, the thymocytes. ATG is used for preventing and treating acute rejection in organ transplantation. ATG acts through four main mechanisms: binding to specific antigens: ATG consists of polyclonal antibodies that recognize various antigens on T and B cells, hence the name “anti-thymocyte”, in particular the T cell receptor and CD3; complement system activation: once ATG binds to target T and B cells, it can activate the complement system, a complex network of blood proteins, that leads to the destruction (lysis) of the targeted cells; phagocytosis: ATG-bound cells are recognized and phagocytized (engulfed) by immune cells such as macrophages, leading to their elimination; and immune response inhibition: beyond direct destruction, ATG also inhibits the function of T and B cells, further suppressing the immune response. ATG does not directly increase inflammatory cytokines but can influence the immune system in ways that may increase susceptibility to infections and inflammation: T cell reduction: ATG destroys thymocytes, reducing the number of circulating T cells. Since T cells regulate immune responses and cytokine production, their depletion alters cytokine balance: compromised immune response: the reduction of T cells and overall immune activity can impair the patient’s ability to mount an immune response. And in regards to adverse reactions, ATG infusion may cause side effects like fever and chills, sometimes associated with a temporary rise in inflammatory cytokines such as interleukin-6 (IL-6) and tumor necrosis factor-alpha (TNF-α). A study published in Transplant Immunology by Beiras-Fernandez et al. showed that ATG induces apoptosis and complement-mediated cell death in peripheral T lymphocytes. It also reduces leukocyte adhesion following ischemia-reperfusion. Moreover, adhesion molecule expression (ICAM-1, VCAM, PECAM, CD11b, and CD62E) was significantly increased in the control group compared to ATG-treated groups. Levels of IL-1, IL-6, and TNF-α were lower in ATG-treated groups than in the control group. Despite these findings, cytokine release syndrome associated with ATG infusion was first described in the 1990s when ATG was introduced as an immunosuppressant in solid organ transplantation. CRS symptoms range from mild systemic effects (fever, fatigue, and headache) to severe systemic inflammatory responses, including cardiovascular collapse, disseminated intravascular coagulation, multi-organ failure, and death. A study by Knaus et al. reported that, according to ASTCT classification, CRS is a common (58%) but mostly mild (92% grade 1–2) complication of ATG therapy for graft-versus-host disease (GVHD) prophylaxis. Even mild CRS led to a pronounced systemic inflammatory response, marked by elevated C-reactive protein (CRP), IL-6, and procalcitonin (PCT) levels [14,19,20,21,22,23,24,25,26,27].

Consequently, transplant recipients are particularly vulnerable to infections in the immediate postoperative period [18].

Early identification of infections in this critical window is crucial, as infections can rapidly escalate to sepsis, a life-threatening condition with a high mortality rate. However, distinguishing between infection and systemic inflammatory response syndrome (SIRS), which often occurs following major surgery such as HTx, is particularly challenging. Both SIRS and infections present with similar clinical symptoms, such as fever, elevated heart rate, and increased white blood cell count, which complicate the diagnosis and delay the initiation of appropriate antimicrobial therapies [24,25].

Traditionally, inflammatory biomarkers, such as CRP and PCT, have been used to detect infections in various clinical settings. CRP is a well-established marker of inflammation and has been widely used to monitor infections. It increases in response to tissue injury or infection, making it a useful but non-specific marker of inflammation. On the other hand, PCT has gained recognition as a more specific marker for bacterial infections and sepsis. Its level increases in response to bacterial infections but not necessarily in viral or non-infectious inflammatory conditions, making it a valuable tool in critical care settings [26].

However, the utility of these traditional markers in the context of heart transplantation is limited. The immunosuppressive therapies that HTx patients receive can obscure the clinical and biological signs of the infection. ATG is widely used as part of induction immunosuppressive therapy to prevent rejection. However, this therapy can also lead to increases in CRP and PCT levels even in the absence of infection. This phenomenon complicates the interpretation of these markers and reduces their reliability in the immediate postoperative period [14,20,21,22,23,27].

In recent years, two additional biomarkers have emerged as potential tools for early detection of infection: Presepsin and IL-6. Presepsin is a soluble CD14 subtype and is released in response to bacterial infections. It has shown promise for distinguishing between sepsis and SIRS, particularly in critically ill patients. IL-6 is a pro-inflammatory cytokine and plays a central role in the acute-phase response to infection and is associated with both systemic inflammation and infection-related complications. These biomarkers could provide more reliable insights into the inflammatory and infectious processes occurring in Htx patients, offering the potential to guide clinical decision-making more effectively [23,28,29,30].

The primary aim of this study was to assess the kinetics of the four inflammatory markers (CRP, PCT, Presepsin, and IL-6) in HTx patients during the immediate postoperative period. By comparing biomarker levels between patients who developed infections and those who did not, we aimed to determine which markers were most reliable for the early detection of infections in this population. This study will help fill a critical gap in the current understanding of infection management in HTx recipients and provide valuable information to improve clinical outcomes.

## 2. Materials and Methods

### 2.1. Study Design and Population

This retrospective observational study was conducted at the Cardiac Surgery Department of Policlinic of Bari, Italy. The study cohort comprised 126 patients who underwent HTx between January 2022 and November 2024. The patients were divided into two groups based on their postoperative infection status: those who developed infections in the early postoperative period (*n* = 26) and those who did not (*n* = 100). The infected group was defined according to strict clinical and microbiological criteria, including the presence of positive blood, urine, or bronchial cultures; radiological evidence of infection; or persistent fever despite antipyretic and corticosteroid therapy. In detail, clinical criteria were the following: persistent fever > 38 °C lasting for more than 48 h; tachycardia with a heart rate > 100 beats per minute (bpm); hypotension (SBP < 90 mmHg); microbiological criteria: positive blood cultures with bacterial growth > 10^3^ colony-forming units per milliliter (CFU/mL), positive urine cultures with bacterial growth > 10^5^ CFU/mL, and positive bronchoalveolar lavage (BAL) cultures with bacterial growth > 10^4^ CFU/mL; and radiological criteria: evidence of pulmonary consolidations on imaging studies (suggestive of pneumonia or other lung infections) and the presence of pleural effusions with radiological features indicative of an infectious process.

The early postoperative period was defined as the first 10 days following HTx, a critical window during which the risk of infection is highest because of the combined effects of surgical trauma, immunosuppressive therapy, and the underlying conditions of the patient.

### 2.2. Inclusion and Exclusion Criteria

The inclusion criteria for this study were as follows: patients who underwent orthotopic HTx between January 2022 and November 2024; patients who were treated with induction immunosuppression using thymoglobulins (rATG); and patients who had postoperative follow-up data available for at least 10 days after the transplant.

Exclusion criteria included the following: patients with pre-existing chronic infections, patients who did not survive the first 48 h after the transplant, and patients who had incomplete medical records. Patients treated with basiliximab or other non-rATG immunosuppressive agents were excluded.

All patients received a standardized immunosuppressive protocol, reducing variability between groups. Ischemic time and cardiopulmonary bypass duration were compared between the two groups. These parameters were included in the statistical models to exclude their confounding effect on the inflammatory response.

This was performed to ensure the reliability of the data and to minimize confounding factors related to long-standing infections or extremely early postoperative mortality.

### 2.3. Data Collection

Data were collected retrospectively from the electronic medical record system of the hospital. Information on patient demographics, preoperative characteristics (including Intermacs classification and the etiology of heart failure), intraoperative variables (such as ischemic time, cardiopulmonary bypass duration, and use of mechanical circulatory support), and postoperative outcomes (ICU length of stay, duration of mechanical ventilation, and in-hospital mortality) were extracted and analyzed.

Inflammatory biomarkers, including CRP, PCT, Presepsin, and IL-6, were measured at predetermined time points: preoperatively and on postoperative days 1, 2, 3, 4, 5, and 10. Biomarker levels were measured using standardized laboratory assays, and the results were stored in a Microsoft Excel^®^ database for analysis. In addition to biomarkers, microbiological data (blood, urine, and bronchial cultures) and radiological findings were reviewed to confirm the presence of infections in the postoperative period.

### 2.4. Inflammatory Biomarker Descriptions

The biomarkers evaluated in this study were selected based on their relevance in detecting infection and potential utility in distinguishing between sterile inflammation and bacterial infection:

- **C-reactive protein (CRP):** CRP is an acute-phase protein produced by the liver in response to systemic inflammation. Its levels increase in response to both infections and non-infectious inflammatory conditions, making it a useful but non-specific marker [23].
- **Procalcitonin (PCT):** PCT is produced in response to bacterial infections, making it more specific for the detection of sepsis and systemic bacterial infections. It is considered superior to CRP in distinguishing bacterial infections from other inflammatory states [21,22,23,27,28,29,30,31].
- **Presepsin:** Presepsin is a soluble form of CD14 released into the bloodstream during bacterial infections as part of the innate immune response. It has shown promise as a marker for sepsis, particularly in critically-ill patients, and is gaining recognition for its ability to distinguish between SIRS and sepsis [23,28,29,30,31,32,33,34,35].
- **Interleukin-6 (IL-6):** IL-6 is a cytokine involved in the regulation of immune and acute-phase inflammatory responses. Elevated levels of IL-6 have been associated with both infection and systemic inflammation; however, its kinetics may provide valuable information on the progression of the inflammatory response in HTx patients [36,37,38,39,40,41,42].

### 2.5. Statistical Analysis

The primary aim of the statistical analysis was to compare the CRP, PCT, Presepsin, and IL-6 levels between the infected and non-infected groups to identify the most reliable biomarkers for detecting early postoperative infections in HTx patients. Continuous variables, such as biomarker levels, were expressed as median values with interquartile ranges (IQR) and compared using the Mann–Whitney U test. Categorical variables, such as the presence of infection and mortality rates, were compared using Fisher’s exact test or the chi-square test, as appropriate.

A logistic regression model with LASSO regularization was employed to assess the predictive value of the biomarkers. Odds ratios (OR) and 95% confidence intervals (CI) were calculated for each variable to identify the independent predictors of infection. Results were considered statistically significant at a *p*-value of less than 0.05. Statistical analyses were performed using Galileo^®^ software 1.5.1.4.2474 and Medicloud^®^ software 1.2.0.568 with additional checks for multicollinearity and robustness of the findings.

## 3. Results

### 3.1. Preoperative Characteristics

The study cohort included 126 HTx patients with a median age of 62 years (IQR: 57–68). The baseline characteristics of the two groups were similar in terms of age and sex distribution, but notable differences emerged when examining the severity of the preoperative conditions. A significantly higher proportion of patients who developed postoperative infections were classified as Intermacs class 1–2, indicating a more severe preoperative condition with advanced heart failure (92.3% vs. 46.0%; *p* = 0.004). Furthermore, a higher percentage of infected patients had acute heart failure onset, which was associated with a more rapid decline in cardiac function than that in patients with chronic heart failure (38.5% vs. 4.0%; *p* = 0.003).

Preoperative CRP levels were also significantly elevated in the infected group compared with those in the non-infected group (133 mg/L vs. 4 mg/L; *p* < 0.001). The main clinical and demographic characteristics of the study population are shown in Table 1.

### 3.2. Intraoperative Variables

Intraoperative variables such as ischemic time and cardiopulmonary bypass duration were comparable between the two groups. The median ischemic time was 240 min in the infected group and 209 min in the non-infected group, with no statistically significant difference (*p* = 0.116). Similarly, the median cardiopulmonary bypass duration was 193 min in the infected group and 179 min in the non-infected group (*p* = 0.281). As shown in Table 2, intraoperative variables such as ischemic time and cardiopulmonary bypass duration were evaluated.

However, a significant difference was observed in the use of short-term mechanical circulatory support (MCS) devices. Patients who developed postoperative infections were more likely to require extracorporeal membrane oxygenation (ECMO) or other forms of MCS (46.2% vs. 14.0%; *p* = 0.030).

### 3.3. Postoperative Outcomes

The postoperative course was significantly more complicated in the infection group. In-hospital mortality was markedly higher among the infected patients than among those who did not develop infections (61.5% vs. 14.0%; *p* = 0.001), with infection-related complications being the primary cause of death in the infected group (46.2% vs. 0%; *p* < 0.001). The median length of stay in the intensive care unit (ICU) was significantly longer for infected patients (23 days vs. 6 days; *p* = 0.001), as was the duration of mechanical ventilation (267 h vs. 29 h; *p* < 0.001).

In addition, patients who developed infections were more likely to require renal replacement therapy (46.2% vs. 8.0%; *p* = 0.003), indicating a severe systemic impact of the infection on organ function in these patients. Postoperative clinical outcomes in infected and non-infected patients are summarized in Table 3.

### 3.4. Lasso Regression

This analysis aimed to identify independent associations with the onset of infection in patients undergoing heart transplantation. The dataset included a series of demographic, clinical, and laboratory parameters. To manage the multicollinearity inherent in the dataset and identify the most relevant predictors, we used a logistic regression model with L1 regularization (Lasso). The logistic regression analysis identifying independent predictors of postoperative infections is presented in Table 4.

Lasso regression adds a penalty equivalent to the absolute value of the magnitude of the coefficients, which aids in feature selection by reducing some coefficients to zero. We extracted non-zero coefficients from the LASSO model to identify variables significantly associated with infection. For each variable, odds ratios (OR) with 95% confidence intervals (CI) and *p*-values were calculated to quantify the strength and significance of the associations.

Lasso regression showed a statistically significant correlation only for preoperative CRP values (*p* = 0.04).

### 3.5. Logistic Regression Analysis (Alternative to LASSO)

We addressed the issue of perfect separation by removing the constant and normalizing the data. The logistic regression model produced the following coefficients:

- Presepsin (POD1): 2.32 → Strong association with infection.
- IL-6 (POD3): 1.02 → Moderate association with infection.
- CRP (POD2): −0.91 → Unexpected negative correlation.
- PCT (POD5): 0.06 → No significant correlation.

The model’s intercept is −2.63, indicating that in the absence of elevated biomarkers, the baseline risk of infection is low.

#### Comparative ROC-AUC Analysis

The AUC results are as follows:

- Presepsin (POD1) = 1.00 → A perfect discriminator, though it requires validation in larger cohorts.
- IL-6 (POD3) = 0.89 → Good discriminative ability but less useful for early detection.
- CRP (POD2) = 0.58 → Poor discrimination, making it an unreliable marker for early infection.
- PCT (POD5) = 0.50 → No discriminative ability, performing no better than random chance.

### 3.6. Inflammatory Biomarker Kinetics

#### 3.6.1. C-Reactive Protein (CRP)

**Figure 1:**
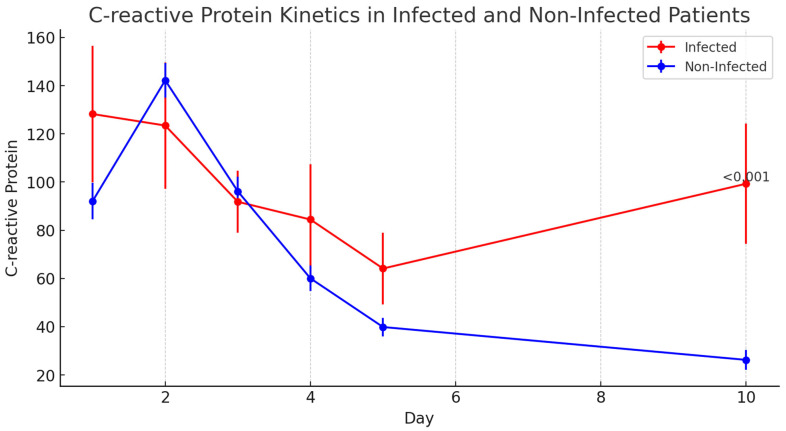
Evolution of C-reactive protein (CRP) levels on postoperative days (POD) 1, 2, 3, 4, 5, and 10 in infected and non-infected patients. Data are represented as median values with interquartile ranges.

CRP levels were significantly elevated in the infected patients only on day 10 postoperatively (*p* < 0.001), with no significant differences observed in the earlier days. The changes in CRP levels over the postoperative days in infected and non-infected patients are depicted in Figure 1.

#### 3.6.2. Procalcitonin (PCT)

**Figure 2:**
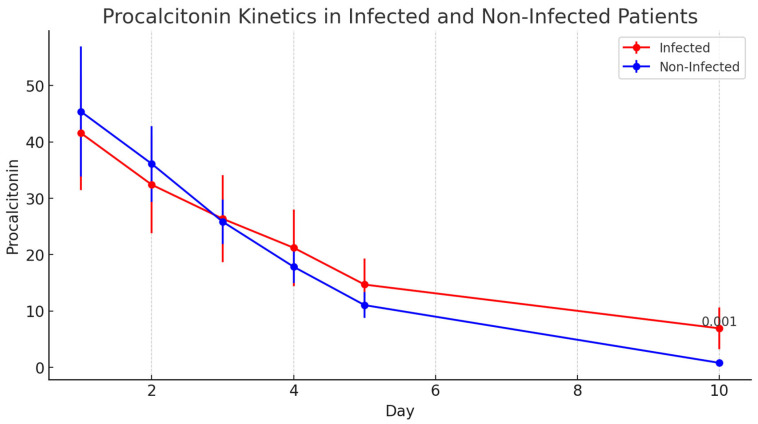
Kinetics of Procalcitonin (PCT) levels over the first 10 postoperative days, comparing infected and non-infected patients.

PCT levels followed a similar trend to CRP levels, with significant differences between the two groups only emerging on day 10 postoperatively (*p* = 0.001), as shown in Figure 2.

#### 3.6.3. Presepsin

**Figure 3:**
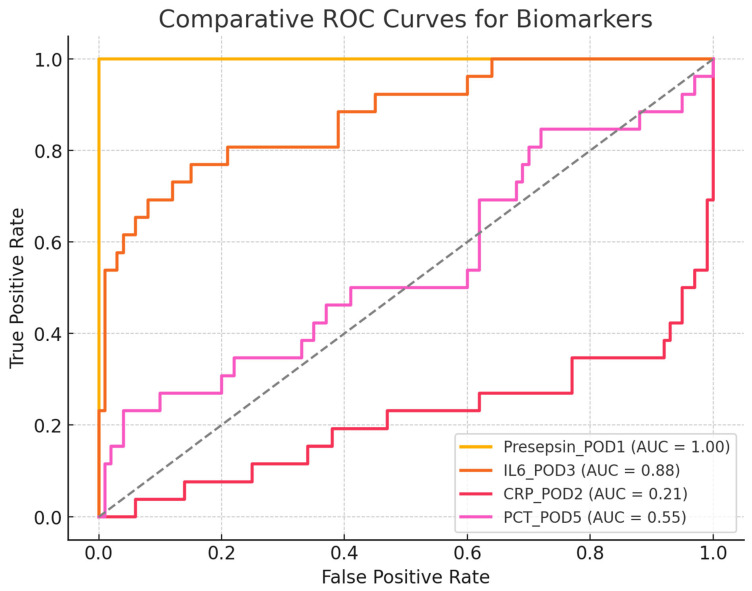
The ROC curves in the graph compare the discriminative ability of Presepsin, IL-6, CRP, and PCT in predicting postoperative infections.

**Figure 4:**
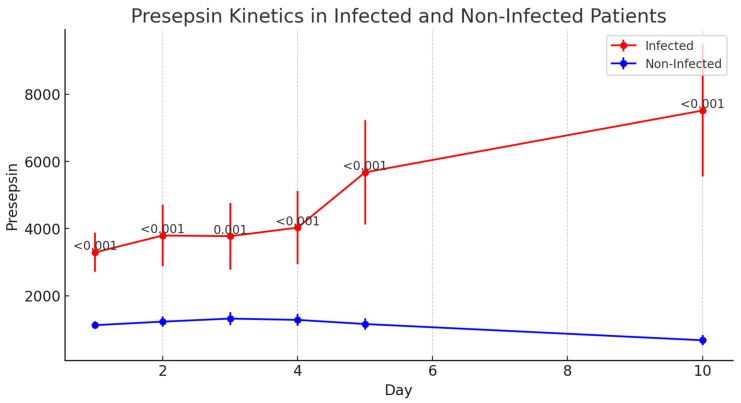
Changes in Presepsin levels over the postoperative period in infected versus non-infected patients, highlighting early elevation in the infected group.

Presepsin levels were significantly elevated in the infected group as early as day 1 postoperatively and remained elevated throughout the 10-day observation period (*p* < 0.001 for all time points). The median Presepsin level on day 1 was 2671 pg/mL in infected patients compared with 907 pg/mL in non-infected patients. The kinetics of inflammatory biomarkers in infected and non-infected patients are presented in Table 5. The evolution of IL-6 levels in the postoperative period, showing statistically significant differences from postoperative day 3 onward, is illustrated in Figure 3. The variation in Presepsin levels in infected versus non-infected patients, highlighting early elevation in the infected group, is shown in Figure 4.

#### 3.6.4. Interleukin-6 (IL-6)

**Figure 5:**
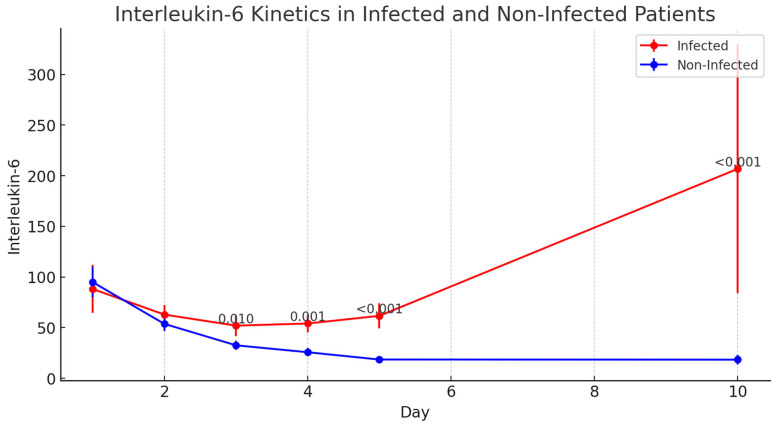
Evolution of Interleukin-6 (IL-6) levels in the postoperative period, showing statistically significant differences from POD 3 onward between infected and non-infected patients.

IL-6 levels showed significant differences between the infected and non-infected groups starting from day 3 postoperatively and continuing through day 10 (*p* < 0.001). The evolution of IL-6 levels in the postoperative period, showing statistically significant differences from postoperative day 3 onward, is illustrated in Figure 5.

The ROC curve shows the discriminative ability of each biomarker to distinguish between infected and non-infected patients.

The AUCs for each biomarker are as follows:

- Presepsin: 1.00 (perfect discriminative ability);
- IL-6: 0.89 (excellent discriminative ability);
- CRP: 0.58 (low discriminative ability);
- PCT: 0.50 (no discriminative ability, equivalent to a random test);
- Chi-square test for AUC comparison:
- Chi-square = 0.90;
- *p*-value = 0.83.

**Figure 6:**
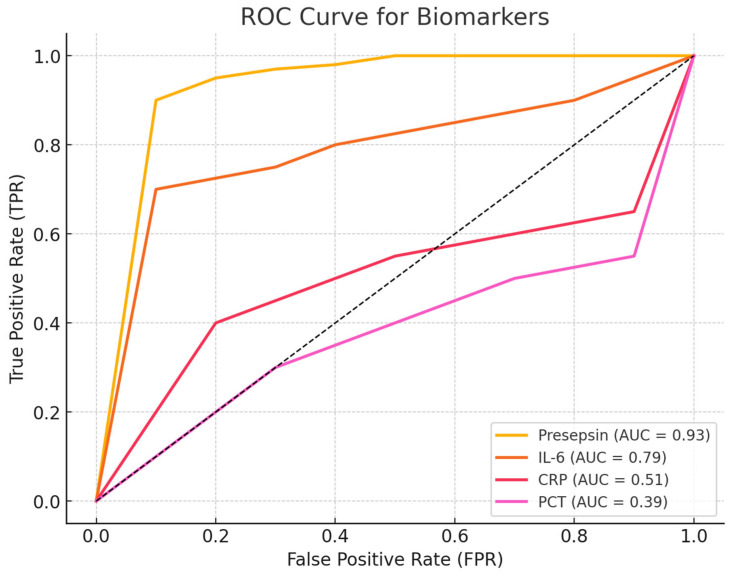
Receiver Operating Characteristic (ROC) curve analysis for CRP, PCT, Presepsin, and IL-6 in distinguishing infected from non-infected heart transplant recipients. The Area Under the Curve (AUC) is provided for each biomarker.

A high *p*-value (>0.05) indicates that the differences between the AUC values were not statistically significant. However, Presepsin and IL-6 had significantly higher AUC values than CRP and PCT levels. Presepsin was the best biomarker, with an AUC of 1.00, suggesting excellent diagnostic capability for identifying post-cardiac transplant infections. IL-6 is also highly useful (AUC = 0.89), whereas CRP and PCT levels do not appear to be reliable for the early diagnosis of infection. Although the differences in AUC values were not statistically significant according to the chi-square test, the practical difference between Presepsin/IL-6 and CRP/PCT is evident and clinically relevant, as shown in Figure 6.

**Figure 7:**
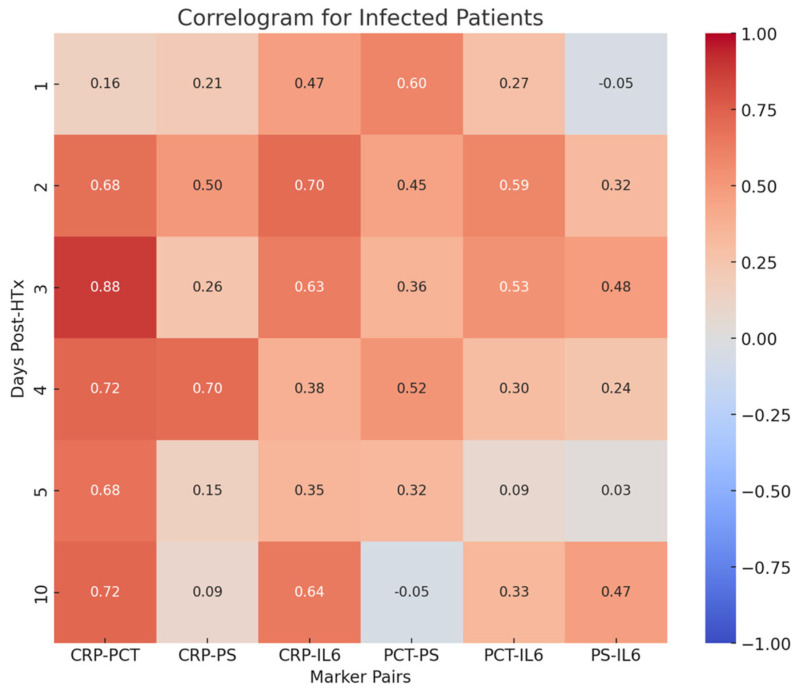
Correlogram illustrating the correlation coefficients between different inflammatory biomarkers in infected and non-infected patients. Significant correlations among biomarkers in infected patients are highlighted.

Correlograms in patients without signs of infection did not show any significant correlations among all the pairs of inflammatory indices examined. A correlogram illustrating correlations between inflammatory biomarkers in infected and non-infected patients is displayed in Figure 7.

The correlogram in patients with signs of infection showed several significant correlations among the pairs of inflammatory indices examined. A correlogram illustrating correlations between inflammatory biomarkers in infected and non-infected patients is displayed in Figure 7:

- PCT-CRP on the third, fourth, and tenth postoperative days (0.88, 0.72, 0.72, respectively);
- CRP-PS on the fourth postoperative day (0.70);
- CRP-IL-6 on the second postoperative day (0.70).

## 4. Discussion

The findings of this study highlight the challenges 438 associated with the use of traditional inflammatory markers, such as CRP and PCT, for 439 the early detection of postoperative infections in HTx patients.

Preoperative CRP levels were significantly elevated in the infected group compared with those in the non-infected group (133 mg/L vs. 4 mg/L; *p* < 0.001). This finding suggests that pre-existing systemic inflammation or infection may predispose patients to develop postoperative infections.

However, a significant difference was observed in the use of MCS devices. Patients who developed postoperative infections were more likely to require ECMO or other forms of MCS (46.2% vs. 14.0%; *p* = 0.030). This finding aligns with previous studies showing that prolonged use of MCS devices is associated with an increased risk of infection due to prolonged exposure to invasive devices and associated risks of nosocomial infections. As shown in Table 2, intraoperative variables such as ischemic time and cardiopulmonary bypass duration were evaluated.

Its delayed response limits the utility of CRP as an early biomarker for detecting infections in HTx patients, suggesting that it may be more useful for monitoring later-stage complications. Although PCT is traditionally viewed as a reliable marker for bacterial infections, its delayed elevation in this study indicates that it may not be an ideal early marker for infection in heart transplant recipients, especially in the context of thymoglobulin-induced immunosuppression. The findings of this study highlight the challenges associated with the use of traditional inflammatory markers, such as CRP and PCT, for the early detection of postoperative infections in HTx patients. Both CRP and PCT levels showed significant differences between the infected and non-infected groups only on postoperative day 10, which may be too late to guide timely clinical interventions. The delayed elevation of these markers suggests that they are more suitable for monitoring late-stage complications than serving as early indicators of infection.

In contrast, Presepsin and IL-6 have proven to be reliable early markers of infection. In particular, Presepsin demonstrated significant differences between the two groups as early as postoperative day 1, making it a valuable tool for the early detection of infections. This early and sustained elevation is particularly important in the context of HTx, where the early identification of infections can have a significant impact on clinical outcomes. Previous studies have similarly highlighted the diagnostic utility of Presepsin in critically ill patients, and our findings extend this utility to the HTx population. Recently, Presepsin has attracted interest among researchers, but its usefulness is still under investigation. Presepsin levels not only are useful in differentiating between sepsis and SIRS, but can also serve as a prognostic tool in bacterial sepsis [19]. sCD14-ST was first studied in 2005 to differentiate patients with sepsis from healthy controls and patients with SIRS [14]. The level of sCD14-ST in subjects with sepsis was significantly higher compared to levels in subjects with SIRS and healthy controls. A meta-analysis on Presepsin showed that it has higher sensitivity and specificity when used as a biomarker for the diagnosis of sepsis [20]. However, another systematic review suggested that it should not be studied as sole index for the diagnosis of bacterial infections and issued a warning [21]. Using it in combination with other biomarkers would be more useful to differentiate sepsis from systemic inflammatory response. Several multicenter and prospective studies have demonstrated that Presepsin levels are significantly higher in patients with bacterial infections. Sensitivity ranged from 70% to 87%, and specificity from 63% to 81%, when cutoff values were between 600 and 864 ng/L [14]. A cutoff value of 600 ng/L failed to distinguish between Gram-positive and Gram-negative infections. However, levels above 946 ng/L were well correlated with Gram-negative bacterial infections. Elevated Presepsin values at ICU admission were associated with acute kidney injury, the need for renal replacement therapy, longer ICU stays, more days on mechanical ventilation, and more days on vasopressors. Serial monitoring of Presepsin levels would be more useful for bedside clinicians to assess the adequacy of antibiotic therapy and thus influence overall outcomes. Studies suggest that a reduction in Presepsin levels by day 7 is strongly correlated with the effectiveness of antibiotic therapy [22]. The various performance efficiency values reported in the literature may be due to heterogeneity among included studies, variations in sepsis criteria, and even the type of sample used (plasma, serum, or whole blood) for Presepsin measurement. Further prospective studies with larger and more diverse populations are needed to establish threshold values for Presepsin for diagnosis and prognosis of bacterial infections. Abdelshafey et al. evaluated Presepsin as a biomarker for sepsis and compared it with SIRS and qSOFA [34]. Biomarkers play a role in decision-making tools for sepsis management but also for antibiotic management, which is extremely essential in this era of growing antibiotic resistance. A literature review on biomarkers in sepsis suggests that nearly 258 biomarkers have been evaluated in different clinical contexts of sepsis [43]. Among these biomarkers, some have become established over time, while others remain in various stages of evolution.

IL-6 showed consistent elevation from day 3 onwards albeit not as early as Presepsin. This cytokine plays a central role in the acute-phase response to infection and inflammation, and its elevation in infected patients suggests that it may be a useful marker for monitoring the progression of inflammatory response. The ability of IL-6 to provide insights into both infection and systemic inflammation makes it a valuable tool for guiding clinical decision making in HTx patients, particularly in the context of the complex interplay between infection, inflammation, and immunosuppression.

### 4.1. Limitations of CRP and PCT

As expected, CRP and PCT levels demonstrated limited utility as early biomarkers for infection in this population. The immunosuppressive effects of rATG likely contribute to the delayed elevation of these markers, as previous studies have shown that thymoglobulins can induce increases in both CRP and PCT levels, even in the absence of infection. Zazula et al. and Brodska et al. reported similar findings in transplant populations, where CRP and PCT levels peaked within the first 24 h post-transplant, complicating their interpretation as reliable indicators of infection [44,45,46].

Despite their limitations as early markers, CRP and PCT may still be valuable in monitoring later-stage complications, particularly when used in conjunction with other clinical and laboratory findings. However, their delayed elevation emphasizes the need for more reliable early markers such as Presepsin and IL-6 to guide clinical interventions during the critical early postoperative period.

### 4.2. IL-6: A Reliable Indicator of Systemic Inflammation

In our study, IL-6 levels were significantly elevated in infected patients starting from day 3 postoperatively, making it a useful marker for identifying patients at risk of infection-related complications. IL-6 is a pleiotropic cytokine that is rapidly produced in response to infections and tissue damage, playing a critical role in initiating the acute inflammatory response, hepatic protein synthesis, and immune system activation (Heinrich et al., 1990 [47]). Because of this, IL-6 serves as one of the earliest detectable markers in systemic bacterial infections [47].

Once synthesized locally at the site of inflammation, IL-6 enters the bloodstream and reaches the liver, where it stimulates the production of acute-phase proteins such as CRP, serum amyloid A (SAA), fibrinogen, and haptoglobin [47]. These proteins are routinely used in clinical practice to assess the severity and progression of infection [47,48].

IL-6 is released in response to pathogen-associated molecular patterns (PAMPs), which are recognized by pathogen recognition receptors (PRRs) such as Toll-like receptors (TLRs), as well as pro-inflammatory signals like TNF-α and IL-1β. Moreover, damage-associated molecular patterns (DAMPs)—produced in sterile inflammatory conditions such as burns or trauma—also promote IL-6 expression [30,48].

For example, a rise in serum IL-6 levels after sterile surgical procedures has been shown to precede the increase of body temperature and acute-phase proteins, highlighting its predictive value even when infection is not yet clinically evident [49]. IL-6 has proven to be a valuable biomarker in guiding therapeutic decisions, particularly regarding the initiation, type, and duration of antibiotic treatment: Elevated IL-6 levels at hospital admission may reflect an active systemic inflammatory response due to bacterial infection (Akira et al., 1993) [49]. A serial decline in IL-6 levels following antibiotic initiation has been correlated with clinical improvement, making IL-6 a potential tool for monitoring treatment effectiveness (Libermann & Baltimore, 1990; Akira & Kishimoto, 1992) [43,50]. As such, IL-6 monitoring can help avoid unnecessary or prolonged antibiotic use, contributing to efforts against antimicrobial resistance [51,52,53].

While IL-6 cannot be used as early as Presepsin, its consistent elevation throughout the postoperative period suggests that it could be valuable for monitoring the progression of the inflammatory response and guiding treatment decisions.

## 5. Future Directions

Although this study provides valuable insights into the utility of Presepsin and IL-6 as early biomarkers for infection in HTx patients, further research is needed to validate these findings in larger patient cohorts. Additionally, future studies should explore the impact of different immunosuppressive regimens on the kinetics of these biomarkers as well as their potential interactions with other clinical and laboratory parameters. Expanding the database and including a control group not treated with thymoglobulin could provide additional insights into the role of immunosuppressive therapies in modulating inflammatory responses in heart transplant patients.

## 6. Conclusions

This study demonstrated that Presepsin and IL-6 are more reliable than CRP and PCT for the early detection of postoperative infections in HTx patients. Presepsin levels were significantly elevated in infected patients as early as day 1 postoperatively, whereas IL-6 levels showed significant differences starting from day 3. These findings suggest that Presepsin and IL-6 could be valuable tools for guiding clinical decision-making in the early postoperative period, when timely intervention is critical for improving patient outcomes.

## 7. Study Limitations

This study had some limitations that should be considered. The limited sample size and retrospective design of this study pose significant constraints. The possibility to generalize the results is restricted with a total of 126 patients enrolled and only 26 infected. A small sample increases the risk of statistical bias, making a prospective study on a larger population necessary to confirm these findings. Additionally, the retrospective nature of the study reduces control over confounding variables and data collection methods. Another limitation is the absence of a control group that did not receive rATG. Without such a comparison, it is difficult to isolate the specific effect of immunosuppression on biomarker levels. Since rATG is known to significantly influence CRP and PCT levels, future studies including a control group without rATG would allow for a more precise assessment of biomarker behavior. Despite the use of logistic regression, certain potential confounding factors were not fully addressed. Variations in surgical strategies, the use of MCS, and differences in immunosuppressive regimens were not deeply explored. Including these elements in future multivariate models could enhance the reliability of the conclusions. Regarding biomarker reliability, Presepsin has shown promise as an early marker, but its levels can also be influenced by non-infectious conditions, such as ischemia-reperfusion injury and post-surgical systemic inflammation. Future studies should focus on strategies to differentiate these effects from those caused by bacterial infections. Similarly, IL-6 levels can be affected by non-infectious inflammatory processes, which reduces its specificity as an infection marker. Another issue is the lack of clearly defined clinical cut-offs and practical application strategies. Although the study supports the use of Presepsin and IL-6 as early biomarkers, no precise thresholds have yet been established to guide clinical interventions. Defining such cut-offs is crucial for integrating these biomarkers effectively into clinical practice. Additionally, the study did not assess the economic impact of implementing Presepsin and IL-6 as routine biomarkers. Given the high costs associated with these analyses, future research should include economic evaluations to determine the feasibility and sustainability of their widespread use. Multicentric studies with larger patient cohorts and prospective designs will be essential to validate the clinical utility of these biomarkers. Such studies will provide a more comprehensive evaluation of their effectiveness across different clinical settings and immunosuppressive regimens.

## Figures and Tables

**Table 1:** Preoperative characteristics of the study population.

Variable	Unit		Overall	Infected	Non-Infected	*p*
Preoperative data						
Population		N, %	126 (100.0)	26 (20.6)	100 (79.4)	-
Age	Years	Median [1–3 IQR]	62 [51–65]	60 [49–64]	63 [52–66]	0.166
Male gender		N, %	96 (76.2)	22 (84.6)	74 (74.0)	0.716
Body mass index	kg/m^2^	Mean ± SD	25.9 ± 3.2	26.2 ± 2.2	25.8 ± 3.3	0.692
Body surface area (DuBois)	m^2^	Mean ± SD	1.86 ± 0.19	1.92 ± 0.18	1.84 ± 0.19	0.224
Heart failure		N, %				
**Intermacs class 1–2**			**70 (55.6)**	**24 (92.3)**	**46 (46.0)**	**0.004**
**HF—acute onset**			**14 (11.1)**	**10 (38.5)**	**4 (4.0)**	**0.003**
Risk factors		N, %				
Hypertension			96 (76.2)	20 (76.9)	76 (76.0)	1.000
Diabetes			24 (19.0)	4 (15.4)	20 (20.0)	1.000
Dyslipidemia			84 (66.7)	16 (61.5)	68 (68.0)	0.912
Extracardiac arteriopathy			42 (33.3)	10 (38.5)	32 (32.0)	0.912
Previous cardiac surgery			32 (25.4)	4 (15.4)	28 (28.0)	0.486
**Renal replacement therapy**			**20 (15.9)**	**12 (46.2)**	**8 (8.0)**	**0.003**
**Admitted to ICU**		**N, %**	**52 (41.3)**	**22 (84.6)**	**30 (30.0)**	**<0.001**
**ICU stay > 96 h**			**48 (38.1)**	**22 (84.6)**	**26 (26.0)**	**<0.001**
Mechanical circulatory support		N, %				
**ECMO**			**26 (20.6)**	**18 (69.2)**	**8 (8.0)**	**<0.001**
**Impella**			**6 (4.8)**	**6 (23.1)**	**0 (0.0)**	**0.007**
**C-reactive protein**	**mg/L**	**Median [1–3 IQR]**	**5.0 [4.0–35.0]**	**133.0 [29.0–174.9]**	**4.0 [4.0–10.6]**	**<0.001**

**Table 2:** Intraoperative variables comparing infected and non-infected patients.

Variable	Unit		Overall	Infected	Non-Infected	*p*
Intraoperative Data						
Duration	Min	Median [1–3 IQR]				
Graft ischemic time			210 [185–240]	240 [190–268]	209 [183–227]	0.116
Cardiopulmonary bypass			182 [162–199]	193 [168–225]	179 [160–198]	0.281
Transfusion		N, %				
Whole blood			116 (92.1)	26 (100.0)	90 (90.0)	0.574
Plasma			122 (96.8)	26 (100.0)	96 (96.0)	1.000
Platelets			44 (34.9)	16 (61.5)	28 (28.0)	0.053
Pharmacological support		N, %				
Epinephrine			124 (98.4)	26 (100.0)	98 (98.0)	1.000
Norepinephrine			122 (96.8)	26 (100.0)	96 (96.0)	1.000
Nitric oxide			122 (96.8)	26 (100.0)	96 (96.0)	1.000
Mechanical circulatory support		N, %				
ECMO			26 (20.6)	12 (46.2)	14 (14.0)	0.030

**Table 3:** Postoperative inflammatory biomarkers (CRP, PCT, Presepsin, and IL-6) with median values and interquartile ranges for infected and non-infected patients on postoperative days 1, 2, 3, 4, 5, and 10.

Variable	Unit		Overall	Infected	Non-Infected	*p*
Postoperative Inflammatory Markers						
C-reactive protein	mg/L	Median [1–3 IQR]				
POD-1			85.7 [53.9–130.5]	89.4 [64.1–132.0]	84.0 [52.8–127.5]	0.502
POD-2			126.7 [103.6–155.6]	104.5 [63.1–132.0]	131.3 [106.9–155.7]	0.079
POD-3			87.9 [64.2–118.4]	83.8 [60.0–125.5]	88.1 [65.1–116.0]	0.616
POD-4			52.2 [34.3–73.2]	58.0 [29.5–129.7]	52.2 [35.4–67.7]	0.659
POD-5			33.4 [23.4–48.2]	41.8 [26.5–91.1]	32.9 [23.1–45.8]	0.245
**POD-10**			**19.8 [10.3–51.6]**	**72.4 [31.5–157.0]**	**15.4 [9.0–30.8]**	**<0.001**
**C-reactive protein—AUC**	**mg/L**	**Mean ± SD**	**5054.6 ± 2343.0**	**1294.1 ± 380.4**	**3715.0 ± 2107.9**	**0.020**
Procalcitonin	ng/mL	Median [1–3 IQR]				
POD-1			24.8 [10.9–57.5]	20.0 [11.0–63.0]	26.1 [11.0–44.2]	0.728
POD-2			18.7 [7.4–45.8]	20.0 [5.7–42.0]	18.0 [7.9–46.4]	0.939
POD-3			15.9 [5.5–34.4]	15.9 [4.1–30.5]	17.3 [5.7–34.8]	1.000
POD-4			9.8 [3.9–26.5]	8.6 [3.0–34.4]	10.1 [3.9–24.6]	0.926
POD-5			5.4 [1.9–12.2]	5.7 [2.2–29.0]	5.3 [1.9–11.7]	0.552
**POD-10**			**0.7 [0.2–1.3]**	**2.7 [0.6–4.6]**	**0.7 [0.2–0.9]**	**0.001**
**Procalcitonin—AUC**	**ng/mL**	**Mean ± SD**	**1616.9 ± 1110.3**	**303.2 ± 149.3**	**1128.2 ± 813.6**	**0.035**
Presepsin	pg/mL	Median [1–3 IQR]				
**POD-1**			**1168 [686–1789]**	**2671 [1894–3123]**	**907 [605–1394]**	**<0.001**
**POD-2**			**1084 [667–1898]**	**2843 [1795–3453]**	**794 [637–1426]**	**<0.001**
**POD-3**			**875 [548–2082]**	**2978 [1739–3453]**	**807 [518–1592]**	**<0.001**
**POD-4**			**842 [496–2331]**	**2512 [2135–3976]**	**677 [456–1868]**	**<0.001**
**POD-5**			**741 [362–2656]**	**3134 [2645–5355]**	**612 [328–1366]**	**<0.001**
**POD-10**			**270 [125–1971]**	**3746 [3147–10,545]**	**174 [112–720]**	**<0.001**
**Presepsin—AUC**	**pg/mL**	**Mean ± SD**	**1255 ± 14,237**	**53,138 ± 16,198**	**56,018 ± 11,820**	**0.732**
Interleukin-6	pg/mL	Median [1–3 IQR]				
POD-1			59.1 [35.5–97.8]	60.6 [55.7–93.1]	57.8 [34.8–98.6]	0.610
POD-2			37.5 [25.0–80.7]	56.1 [33.2–89.3]	32.1 [22.4–68.8]	0.091
**POD-3**			**22.9 [18.4–45.2]**	**35.1 [27.1–61.5]**	**20.5 [15.6–33.4]**	**0.010**
**POD-4**			**18.3 [12.5–45.6]**	**48.9 [27.1–77.8]**	**16.6 [11.2–28.1]**	**0.001**
**POD-5**			**13.8 [9.4–37.5]**	**51.3 [37.8–86.1]**	**12.8 [8.6–18.2]**	**<0.001**
**POD-10**			**8.5 [6.5–24.5]**	**63.7 [25.0–112.0]**	**8.0 [6.1–14.3]**	**<0.001**
Interleukin-6—AUC	pg/mL	Mean ± SD	3349.1 ± 1677.2	987.5 ± 591.5	1999.7 ± 1476.0	0.150

**Table 4:** Results of Lasso logistic regression analysis identifying independent predictors of postoperative infections. Variables with significant odds ratios (OR) and confidence intervals (CI) include preoperative CRP, bleeding requiring surgical revision, and prolonged ICU stay before transplantation.

Variable	Coefficient	OR	Lower CI	Upper CI	*p*
Preoperative C-reactive protein	1.05	2.85	1.03	7.87	0.04
Bleeding requiring surgical revision	0.95	2.57	0.93	7.11	0.07
Preoperative ICU stay >96 h	0.51	1.67	0.6	4.61	0.32
Interleukin-6 (POD5)	0.46	1.58	0.57	4.36	0.38
Presepsin (POD1)	0.33	1.38	0.5	3.82	0.53
Presepsin (POD5)	0.15	1.16	0.42	3.2	0.78
Graft ischemic time	0.12	1.12	0.41	3.1	0.82
HF—acute onset	−0.07	0.93	0.34	2.58	0.9
Interleukin-6 (POD2)	−0.12	0.89	0.32	2.46	0.82
Interleukin-6 (POD1)	−0.37	0.69	0.25	1.91	0.48
Age	−0.60	0.55	0.2	1.52	0.25
C-reactive protein (POD2)	−0.61	0.54	0.2	1.5	0.24

**Table 5:** Postoperative clinical outcomes in infected and non-infected patients.

Variable	Unit	Overall		Infected	Non-Infected	*p*
Postoperative Data						
**In-hospital mortality**		**N, %**	**30 (23.8)**	**16 (61.5)**	**14 (14.0)**	**0.001**
Cause of death		N, %				
Cardiac			8 (6.3)	2 (7.7)	6 (6.0)	1.000
**Infection-associated**			**12 (9.5)**	**12 (46.2)**	**0 (0.0)**	**<0.001**
Duration		Median [1–3 IQR]				
**ICU-stay**	**Days**		**6 [5–18]**	**23 [12–37]**	**6 [5–8]**	**0.001**
Hospital stay	Days		31 [26–98]	44 [23–56]	28 [23–37]	0.333
**Mechanical ventilation**	**Hours**		**31 [26–98]**	**267 [99–360]**	**29 [24–50]**	**<0.001**
Mechanical circulatory support		N, %				
ECMO			34 (27.0)	14 (53.8)	20 (20.0)	0.036
Stroke		N, %				
Ischemic			2 (1.6)	2 (7.7)	0 (0.0)	0.206
Hemorragic			0 (0.0)	0 (0.0)	0 (0.0)	1.000
**Renal replacement therapy**		**N, %**	**62 (49.2)**	**24 (92.3)**	**38 (38.0)**	**<0.001**
**Bleeding requiring surgical revision**		**N, %**	**18 (14.3)**	**12 (46.2)**	**6 (6.0)**	**0.002**
Transfusion		N, %				
Whole blood			118 (93.7)	26 (100.0)	92 (92.0)	0.572
Plasma			122 (96.8)	26 (100.0)	96 (96.0)	1.000
**Platelets**			**44 (34.9)**	**16 (61.5)**	**28 (28.0)**	**0.05**

## Data Availability

The data presented in this study are available upon request from the corresponding author.

## Abbreviations

The following abbreviations are used in this manuscript:

HTx	Heart Transplantation
CRP	C-reactive Protein
PCT	Procalcitonin
IL-6	Interleukin-6
SIRS	Systemic Inflammatory Response Syndrome
rATG	Rabbit Anti-Thymocyte Globulin
POD	Postoperative Day
ICU	Intensive Care Unit
ECMO	Extracorporeal Membrane Oxygenation
MCS	Mechanical Circulatory Support
IQR	Interquartile Range
OR	Odds Ratio
CI	Confidence Interval
ROC	Receiver Operating Characteristic
AUC	Area Under the Curve
HF	Heart Failure
BMI	Body Mass Index
SD	Standard Deviation
LASSO	Least Absolute Shrinkage and Selection Operator
DNA	Deoxyribonucleic Acid
RNA	Ribonucleic Acid
